# Supplementary material for: Pulsed axial epitaxy of colloidal quantum dots in nanowires enables facet-selective passivation
Source: Nat Commun. 2018 Nov 23;9:4947. doi: 10.1038/s41467-018-07422-4 (PMC6251926; doi:10.1038/s41467-018-07422-4)
Supplement: Supplementary file 1 — Supplementary Information [file 41467_2018_7422_MOESM1_ESM.pdf]

Supplementary Information

Pulsed axial epitaxy of colloidal quantum dots in nanowires enables facet-selective passivation

Li et al.

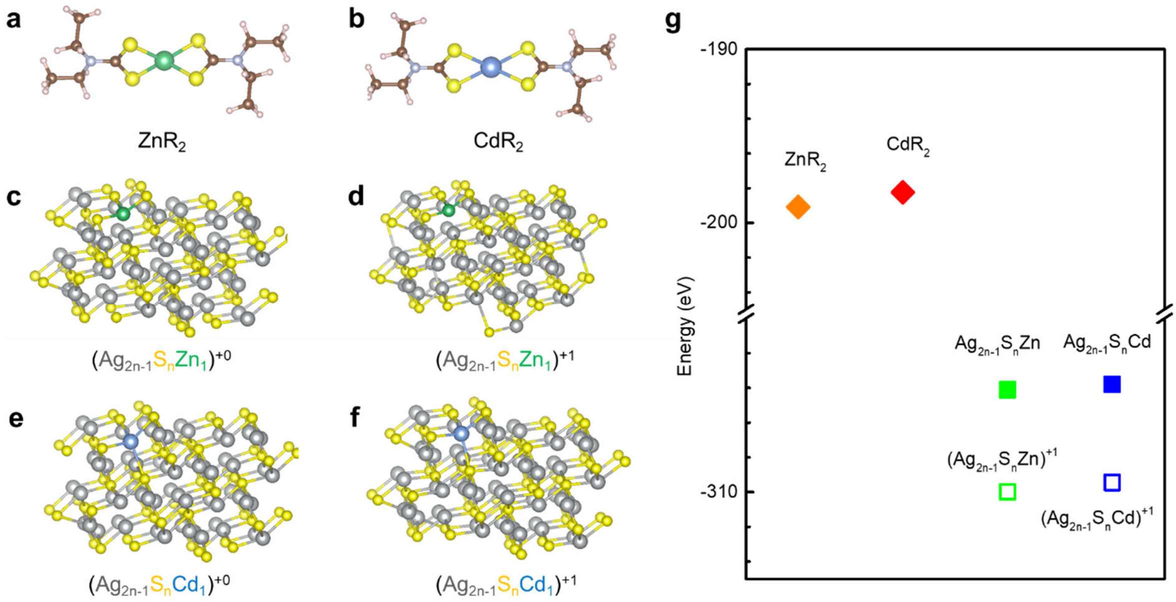

Supplementary Figure 1 | DFT calculations on the relative intercalation energy. a,**b**, Structures of Zn precursors (Zinc diethyldithiocarbamate, denoted as ZnR_2) and Cd precursors (Cadmium diethyldithiocarbamate, denoted as CdR_2), respectively. **c**,**d**, Neutral (**c**) and charged (**d**) insertion of Zn into Ag_2S catalyst, in the form of $(Ag_{2n-1}S_nZn_1)^{+0}$ and $(Ag_{2n-1}S_nZn_1)^{+1}$, respectively. **e**,**f**, Neutral (**e**) and charged (**f**) insertion of Cd into Ag_2S catalyst, in the form of $(Ag_{2n-1}S_nCd_1)^{+0}$ and $(Ag_{2n-1}S_nCd_1)^{+1}$, respectively. **g**, Calculated energies for different species.

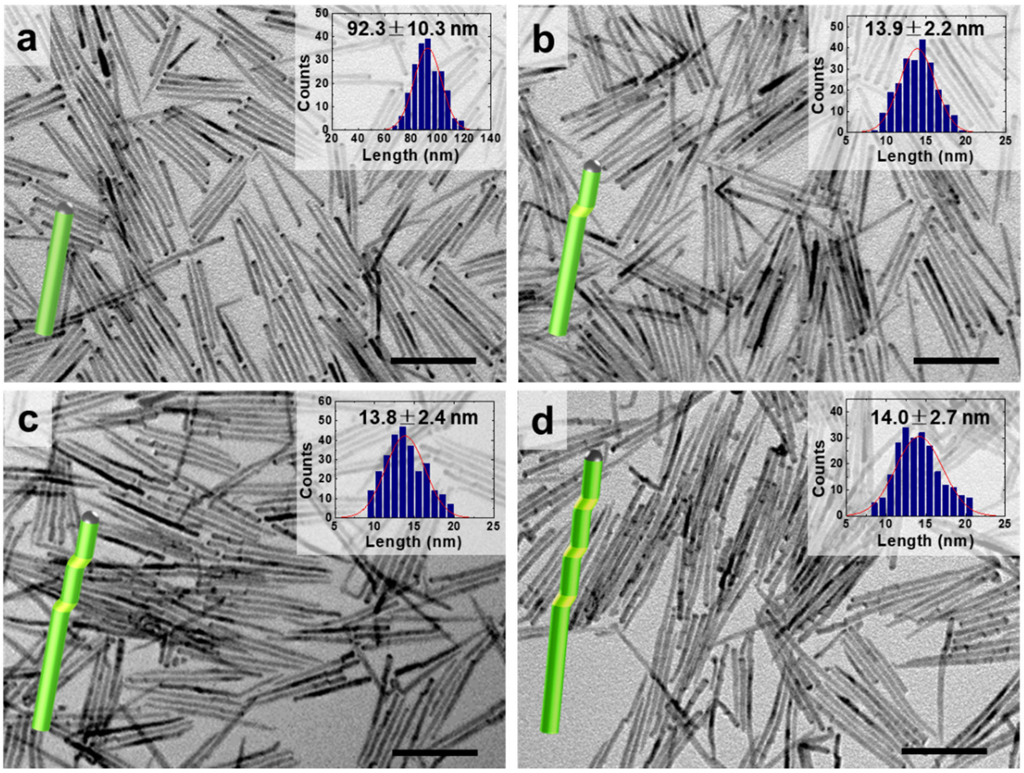

Supplementary Figure 2 | Low magnification TEM images of CdS-ZnS QDNWs. a, ZnS NRs. **b**, 1CdS-2ZnS QDNWs. **c**, 2CdS-3ZnS QDNWs. **d**, 3CdS-4ZnS QDNWs. Insets are corresponding statistical length distribution of each newly epitaxial ZnS segment. Scale bars are 100 nm for **a-d**.

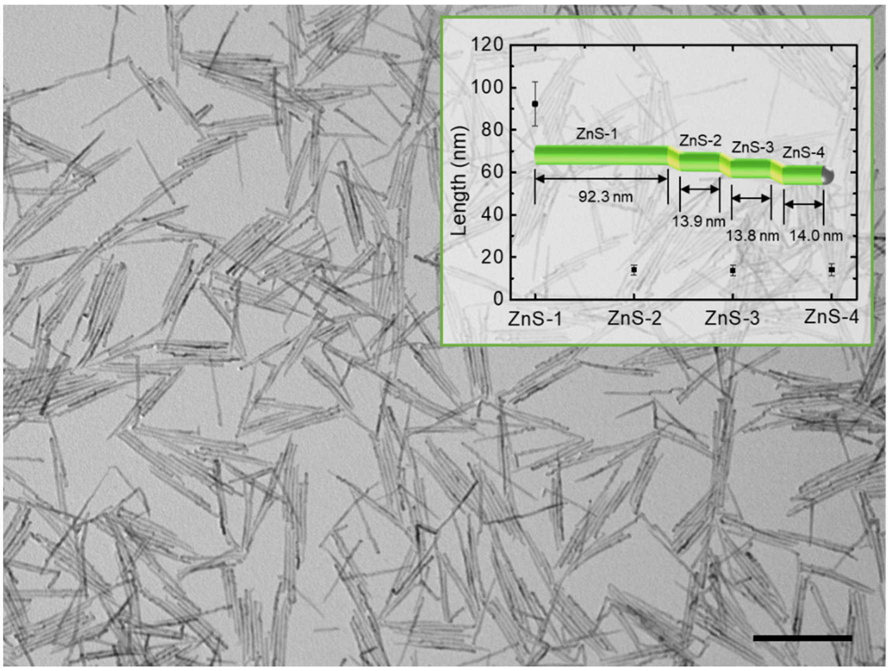

Supplementary Figure 3 | Large-area TEM image of 3CdS-4ZnS QDNWs. Inset is the corresponding statistical length evolution of each newly epitaxial ZnS segment. The scale bar is 100 nm.

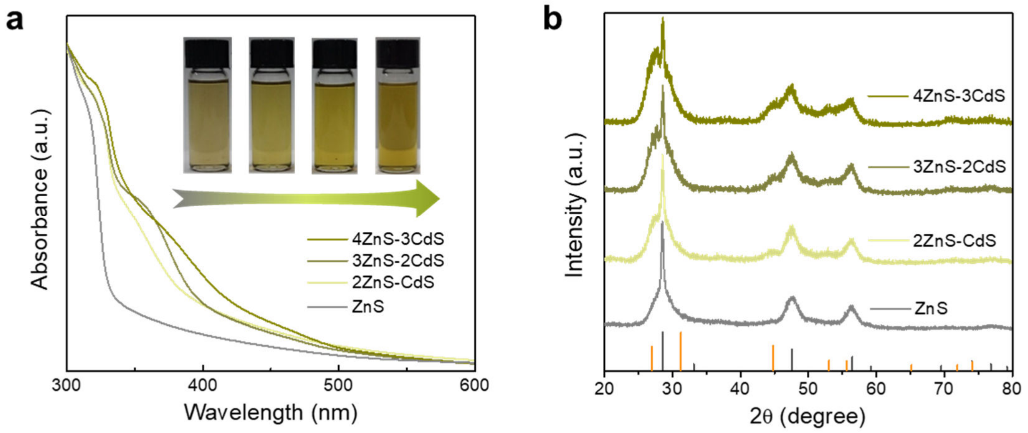

Supplementary Figure 4 | Spectra and X-ray diffraction patterns of CdS-ZnS QDNWs. a, UV-Vis absorption spectra of as-prepared nanowires. Inset are optical photographs of these QDNWs with gradual color change due to CdS insertion. **b**, Powder X-ray diffraction (PXRD) patterns can be unambiguously indexed to cubic zinc-blende (ZB) ZnS (JCPDS No. 65-0309) and ZB CdS (JCPDS No. 65-8873).

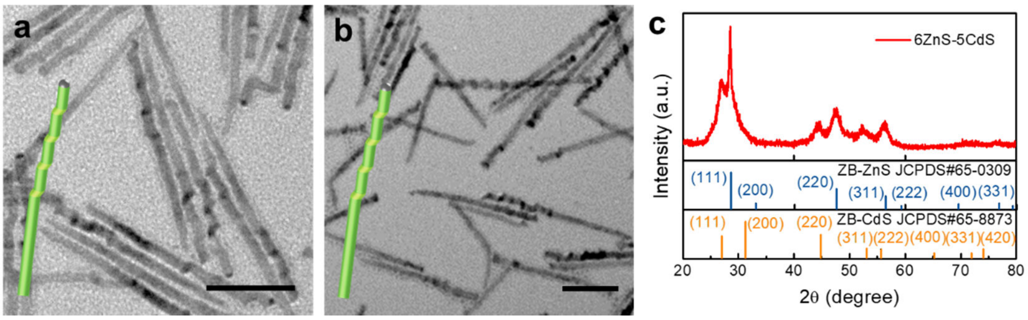

Supplementary Figure 5 | Characterization of QDNWs with controllable 4 and 5 CdS QDs stacked in the ZnS nanowires. a, TEM image of 4CdS-5ZnS QDNWs. **b**, TEM image of 5CdS-6ZnS QDNWs. **c**, PXRD pattern of 5CdS-6ZnS nanowires with indexed crystalline structure. Scale bars are 50 nm for **a** and **b**.

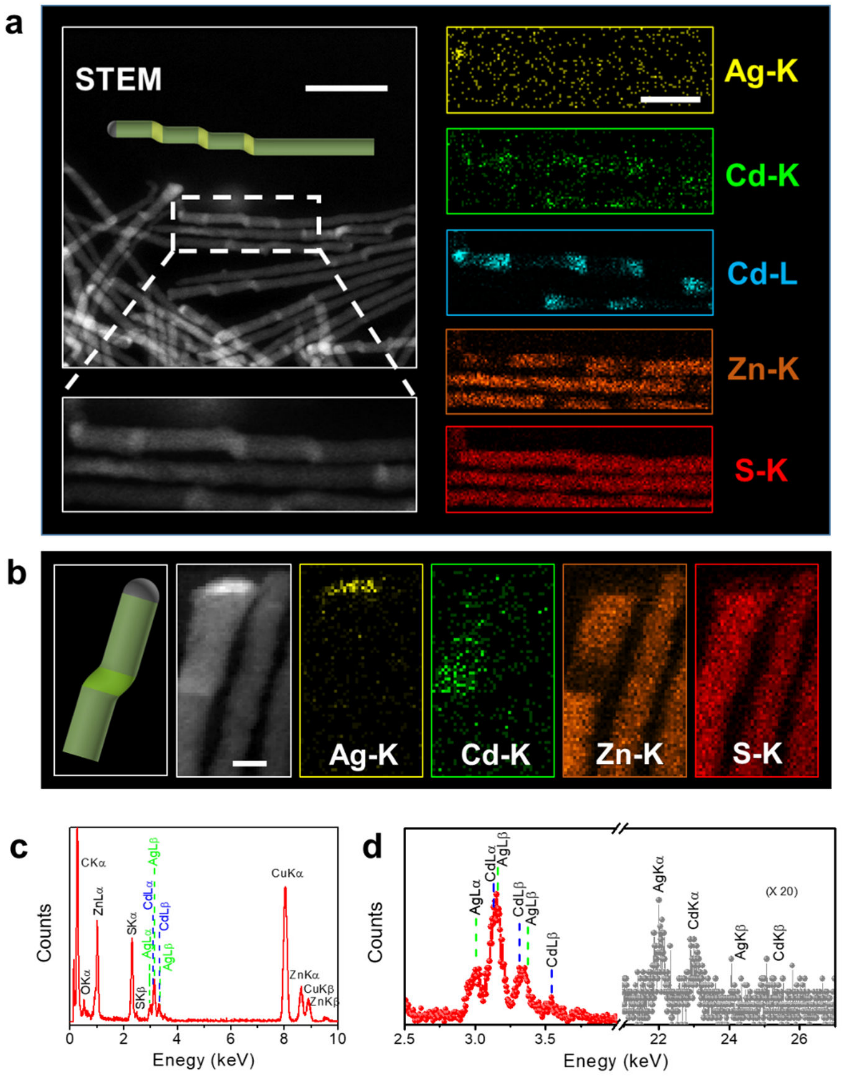

Supplementary Figure 6 | EDS analyses of QDNWs. a,**b**, Dark-field STEM images and EDS elemental mapping images of 3CdS-4ZnS QDNWs. **c**,**d**, Cd-K and Cd-L signals are combined together to demonstrate the actual distribution of CdS component, due to the strong but indistinguishable Ag-L and Cd-L signals as well as the weak but distinguishable Ag-K and Cd-K signals. Scale bars are 50 nm for **a** (left panel), 20 nm for **a** (right panel), and 5 nm for **b**, respectively.

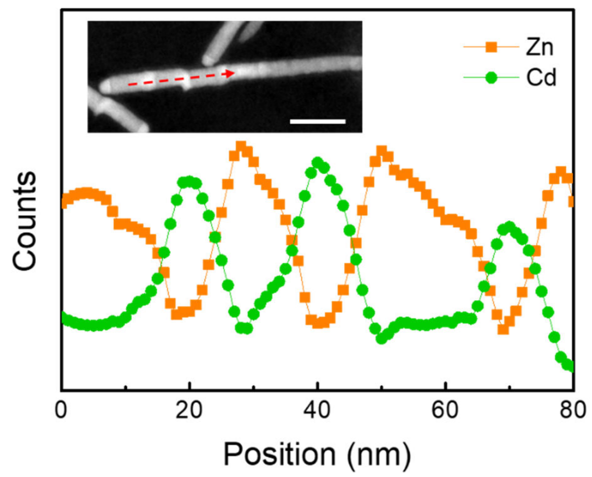

Supplementary Figure 7 | Additional dark-field STEM image and EDS line-scan profiles of 3CdS-4ZnS QDNWs. They show clear alternate distributions of ZnS and CdS segments. The scale bar in inset is 50 nm.

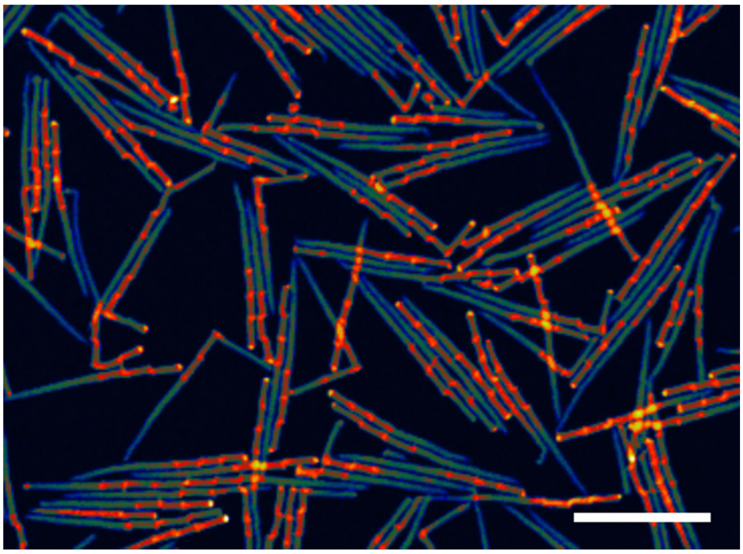

Supplementary Figure 8 | Large-area STEM image of 3CdS-4ZnS QDNWs with high-degree uniformity of the dotted structure. Scale bar, 100 nm.

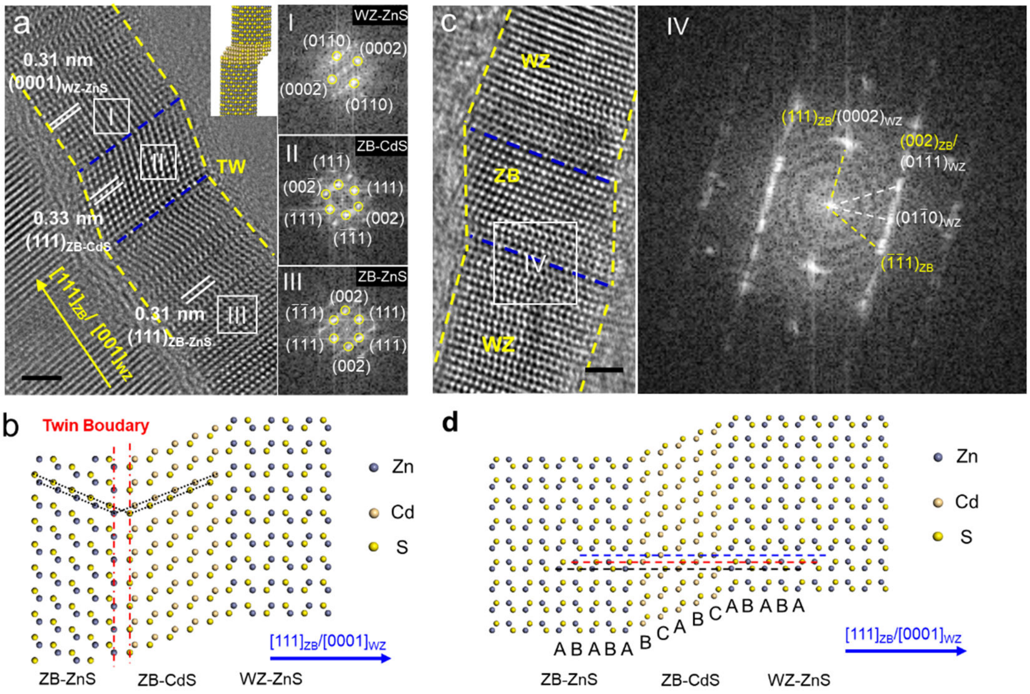

Supplementary Figure 9 | Two epitaxy modes. a,**b**, HRTEM image (**a**) and corresponding crystal structure model (**b**) of the CdS-ZnS QDNWs, featuring a ZB-ZB(TW)-WZ epitaxial mode along the growth direction. **c**,**d**, HRTEM image (**c**) and corresponding crystal structure model (**d**) of another CdS-ZnS QDNWs, featuring a WZ-ZB-WZ epitaxy mode along the growth direction in the ABABA|BCABC|ABAB stacking sequence. Insets in **a** and **c** are corresponding fast Fourier transformed (FFT) images of the selected HRTEM areas. Scale bars are 2 nm for **a** and **f**.

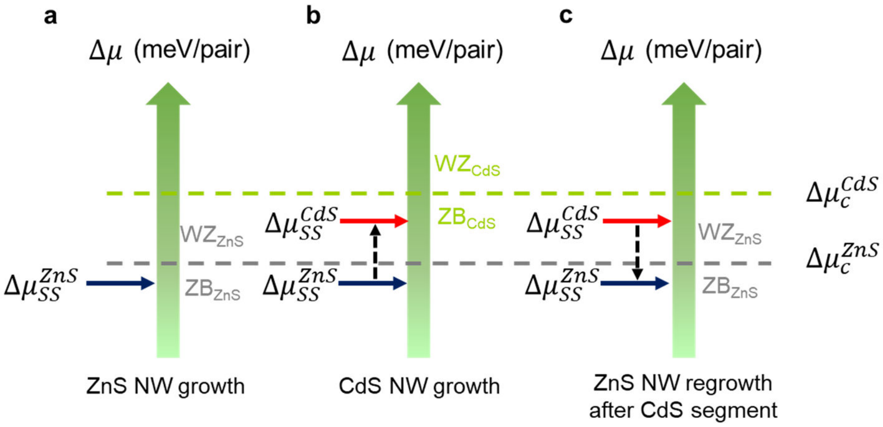

Supplementary Figure 10 | Schematic of the possible mechanism for the observed phase transitions in CdS-ZnS QDNWs. The possible configurations of time-dependent supersaturation $\Delta\mu_{SS}$ and material-dependent critical supersaturation $\Delta\mu_{c}$ during ZnS NW growth (**a**), CdS QD growth after addition of Cd precursors (**b**), and ZnS regrowth just after the completion of CdS growth (**c**).

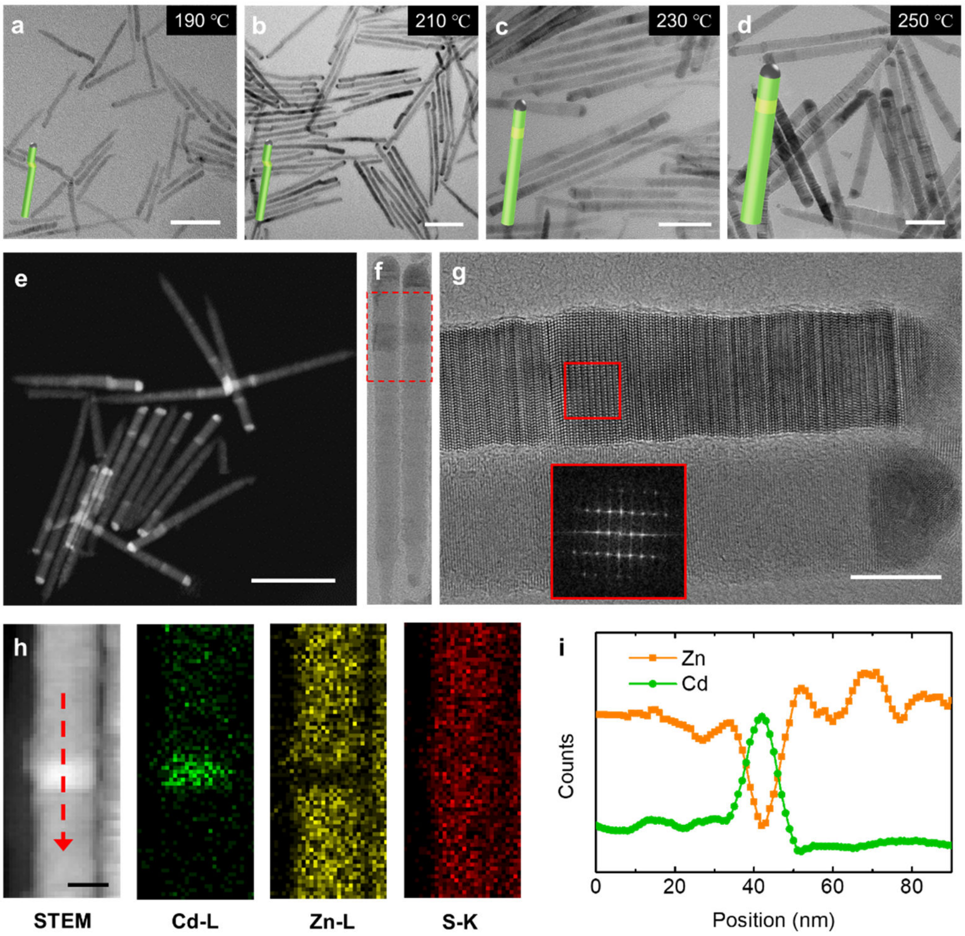

Supplementary Figure 11 |TEM images of CdS-ZnS QDNWs grown at different temperatures. a-d, Low-magnification TEM images of CdS-ZnS QDNWs grown at temperatures of 190 °C, 210 °C, 230 °C, and 250 °C, respectively. **e**, Dark field STEM images of CdS-ZnS (250 °C) nanowires with more straight CdS segments, and average sizes of CdS segment are 10 nm in length and 12 nm in diameter. **f-g**, HRTEM image (**g**) of CdS-ZnS (250 °C) nanowires in the red dash boxes in **f**, indicating that the straight CdS segment sandwiched in two ZnS segments behaves wurtzite structure when the reaction temperature was elevated to 250 °C. This could be explained with the mechanism in **Supplementary Fig. 10**: higher supersaturation of Cd in catalyst leads to more WZ structure in CdS segments. **h**, EDS elemental mapping images of CdS-ZnS QDNWs grown at 250 °C, shown with Cd-L (green), Zn-L (yellow), and S-K (red) signals. **i**, EDS line-scan profile along the red dash line in **h**. CdS QD is stacked in the ZnS nanowire with clear interface. Scale bars are 50 nm for **a-e**, and 10 nm for **g** and **h**, respectively.

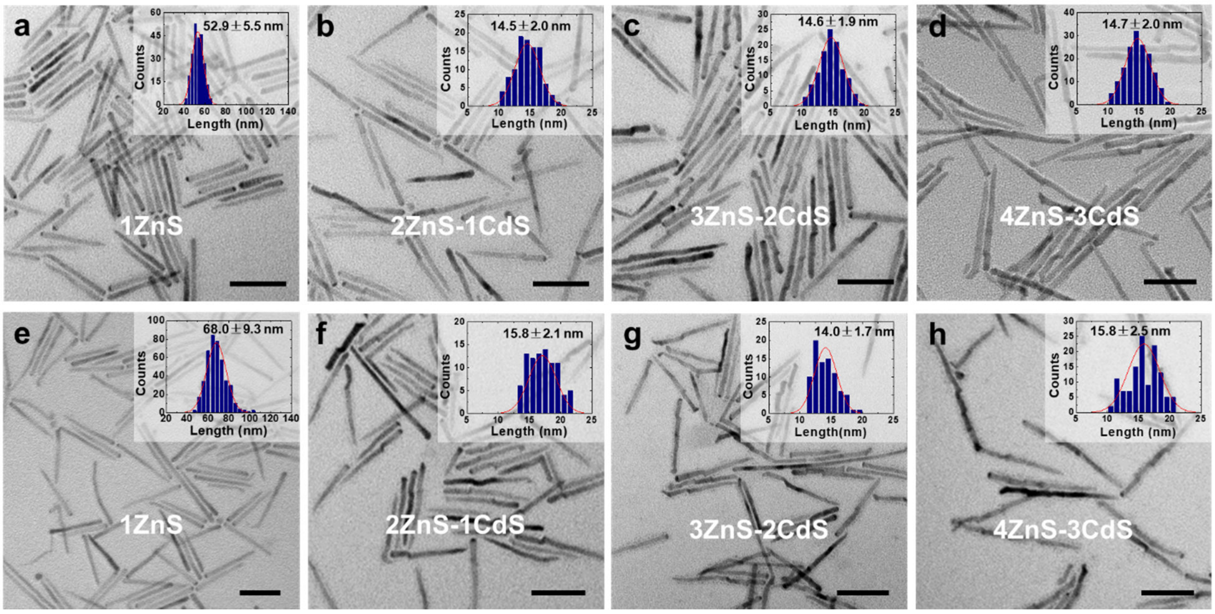

Supplementary Figure 12 | Time-dependent synthesis of CdS-ZnS QDNWs with different initial ZnS growth time. a-d, TEM images of nanowires obtained at different reaction time of 30 min, 50 min, 70 min, 90 min, respectively, with the initial ZnS growth time of 30 min. **e-h**, TEM images of nanowires obtained at different reaction time of 40 min, 60 min, 80 min, 100 min, respectively, with the initial ZnS growth time of 40 min. Insets: corresponding statistical length distributions of each newly epitaxial ZnS segment. Scale bars are 50 nm for **a-h**.

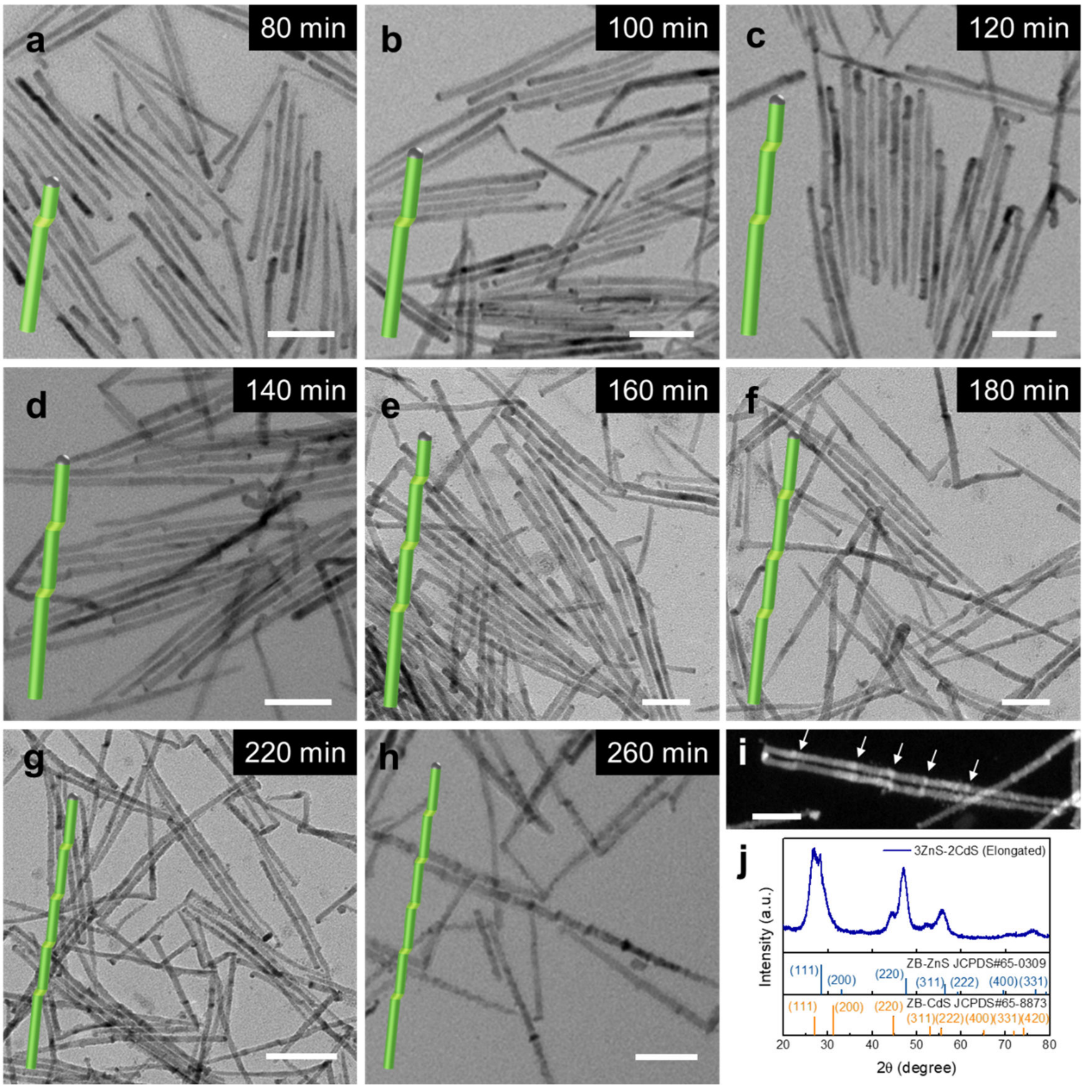

Supplementary Figure 13 | Synthesis of CdS-ZnS QDNWs with elongated ZnS segment through alternate addition of Zn and Cd precursors. a-h, TEM images of ZnS-elongated QDNWs obtained at different reaction time of 80 min, 100 min, 120 min, 140 min, 160 min, 180 min, 220 min, 260 min, respectively, with the initial ZnS growth time of 60 min. Zn and Cd precursors were added alternately at the time interval of 20 min. **i**,**j**, Dark-field STEM image (**i**) and the corresponding PXRD pattern (**j**) of the ZnS-elongated QDNWs in **h**. Each newly grown ZnS segment is generally twice longer than the CdS segment. Scale bars are 50 nm for **a-f**, **h**, and **i**, and 100 nm for **g**, respectively.

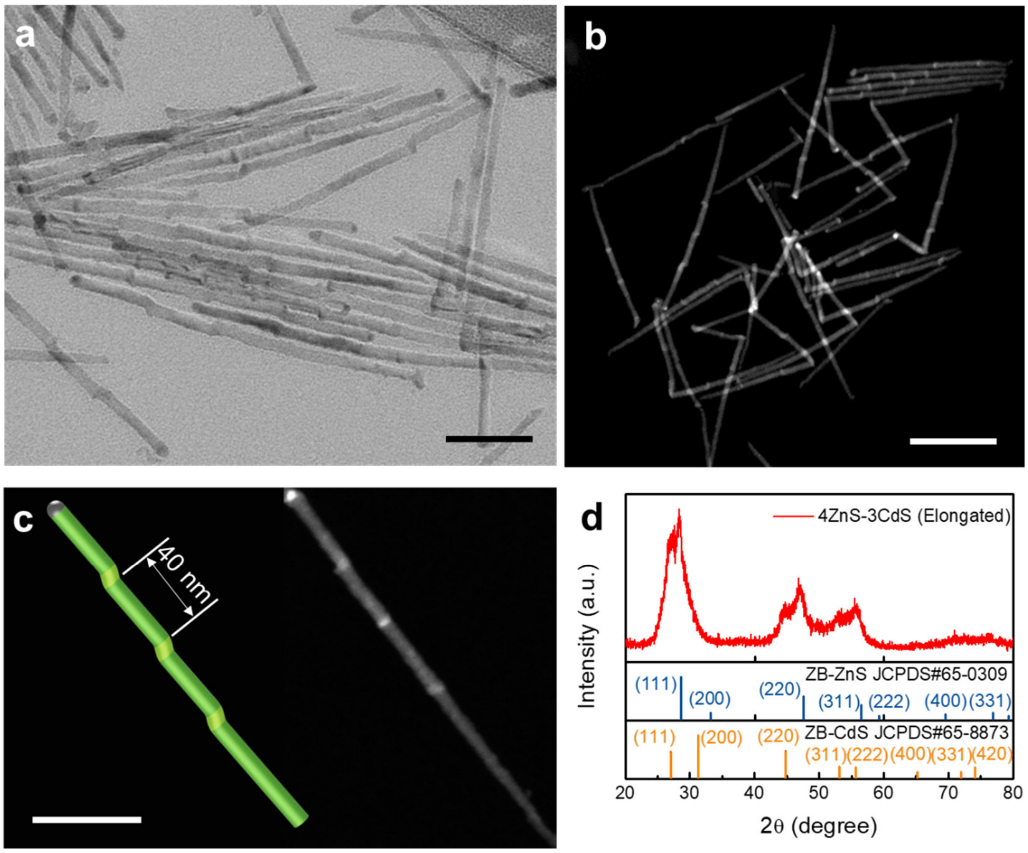

Supplementary Figure 14 | Morphology and structure characterization for ZnS-elongated 3CdS-4ZnS QDNWs. a, **b**, TEM image (**a**) and dark-field STEM image (**b**) of ZnS-elongated QDNWs (**Supplementary Fig. 13f**) obtained at reaction time of 180 min, with the initial ZnS growth time of 60 min. Generally, three CdS QDs with high contrast could be easily identified in the ultrathin heteronanowire. **c**, Geometrical model (left panel) and corresponding dark-field STEM image (right panel) of a typical ZnS-elongated QDNW with average ZnS length of 40 nm. **d**, PXRD pattern of the ZnS-elongated QDNWs. Scale bars are 50 nm for **a** and **c**, and 100 nm for **b**, respectively.

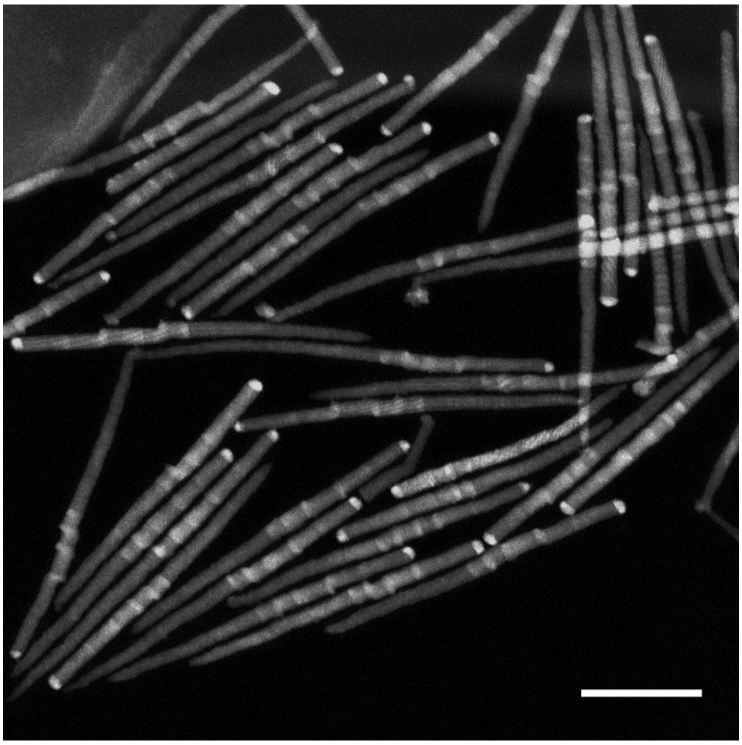

Supplementary Figure 15 | Large-area STEM image of quantum-dots-in-nanowire with gradient spacings between adjacent CdS QDs. Scale bar, 50 nm.

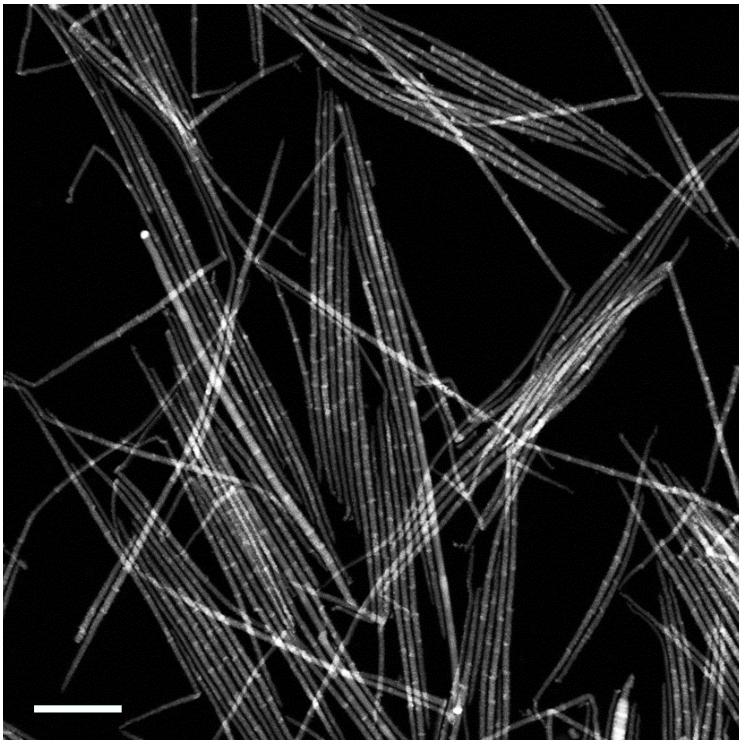

Supplementary Figure 16 | Large-area STEM image of quantum-dots-in-nanowire with 10 CdS QDs stacked in each ZnS nanowire. Scale bar, 100 nm.

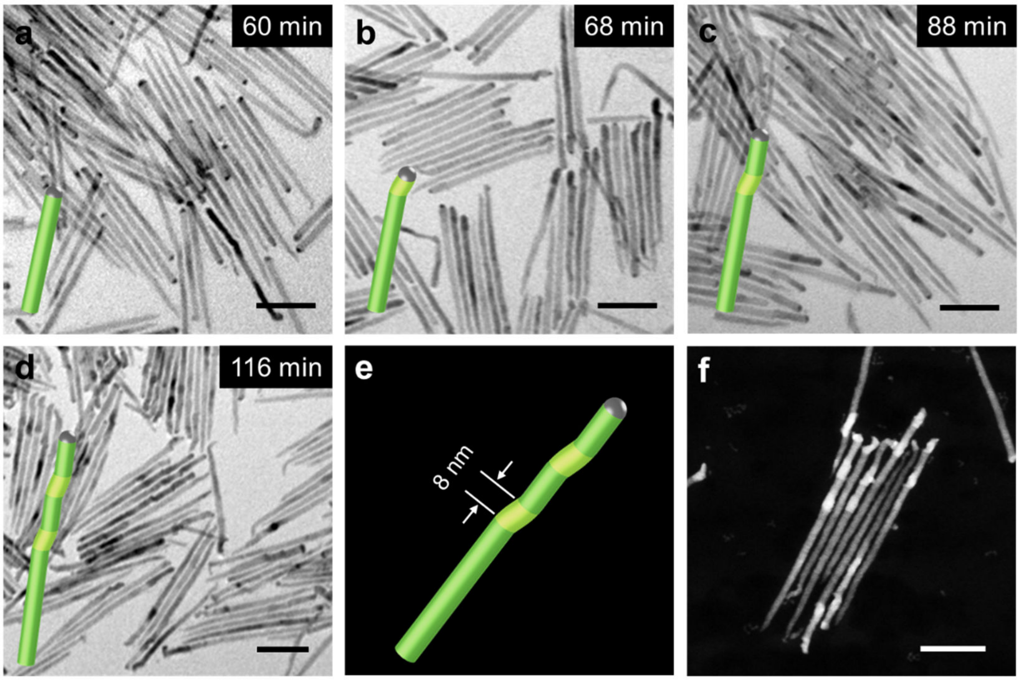

Supplementary Figure 17 | Synthesis of CdS-elongated CdS-ZnS QDNWs. a-d, TEM images of CdS-elongated QDNWs obtained at different reaction time of 60 min, 68 min, 88 min, 116 min, respectively, with the initial ZnS growth time of 60 min. The CdS segment was elongated through twice addition of Cd precursor every 4 min and again 20 min later. **e**, Geometrical model of CdS-elongated 2CdS-3ZnS nanowires with average CdS length of 8 nm. **f**, Corresponding dark-field STEM image of CdS-elongated 2CdS-3ZnS nanowires in **d** and **e**. Scale bars are 50 nm for **a-d** and **f**.

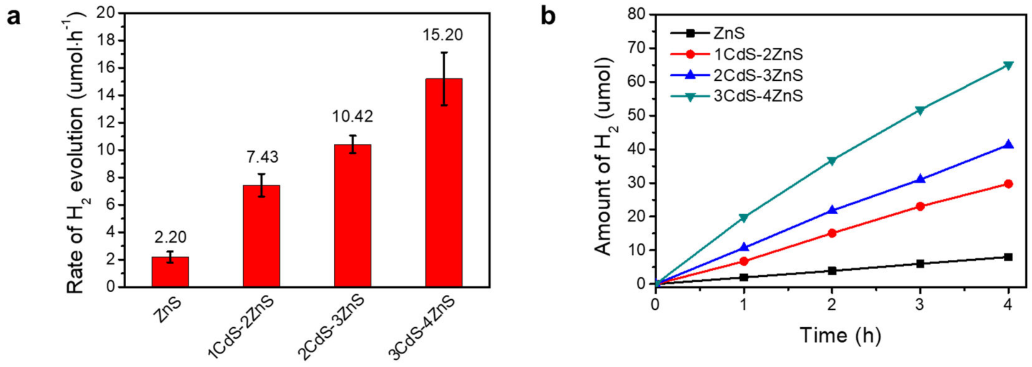

Supplementary Figure 18 | Photocatalytic performances of all QDNWs. Photocatalytic H_2 production rate (**a**) and time-dependent H_2 productions (**b**) of different Ag_2S-tipped QDNWs (20 mg for each sample). Error bars in **a** correspond to the standard deviation values of the H_2 production rates.

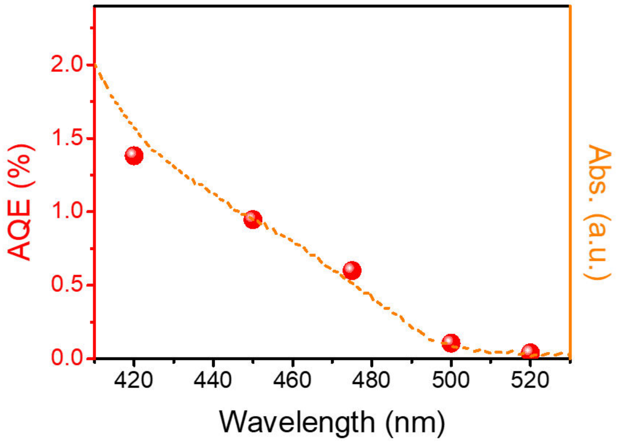

Supplementary Figure 19 | Photocatalytic efficiency of QDNWs. Apparent quantum efficiency (AQE, red dots) in photocatalytic H_2 production and the absorption spectrum (yellow dashed curve) of the 3CdS-4ZnS QDNWs.

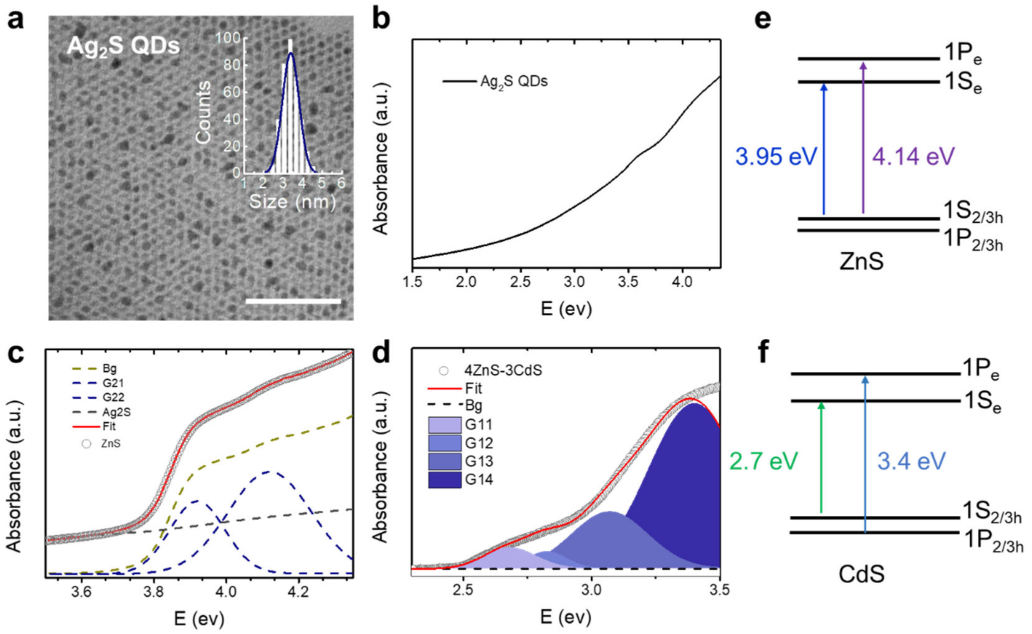

Supplementary Figure 20 | Determination of band structure in CdS-ZnS. a, TEM image of the Ag_2S QDs with a similar size to the Ag_2S tips in CdS-ZnS QDNWs. Inset: size distribution of Ag_2S QDs, averaging at 3.4 ± 0.4 nm. The scale bar is 50 nm. **b**, UV-vis absorption spectrum of the Ag_2S QDs. **c**, Fitted absorption spectra of ZnS NRs by subtracting the absorption of Ag_2S tips in a reported method [1-3]. **d**, Fitted absorption spectra of 3CdS-4ZnS QDNWs by subtracting the absorption of Ag_2S tips. The size of CdS QDs along c-axis is within the exciton Bohr diameter of bulk CdS[4] (ca. 5.5 nm) and the excitonic band structure is very similar with CdS QDs of similar size[2-4]. **e**, **f**, Schematic band structures of ZnS (**e**) and CdS (**f**) components, respectively. Note that, the weak and broad exciton absorption peaks may arise from two main reasons: (1) The dielectric constant of oleic acid, ZnS, and CdS are 2.46, 8.32, and 9.02, respectively. A decreased dielectric contrast (wrapped by ZnS instead of pure organic ligands) will relax the dielectric confinement effect, which influences the exciton binding energy and oscillation strength[4-9], (2) Inhomogeneous broadening due to size distributions.

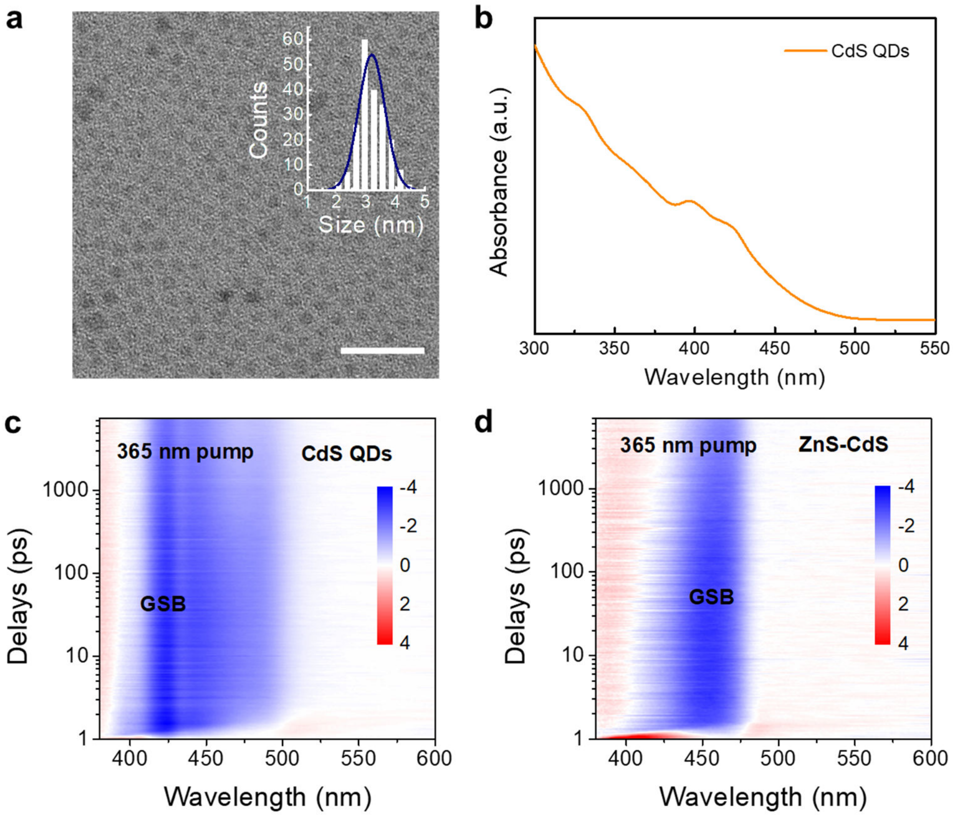

Supplementary Figure 21 | Spectral characterizations of CdS QDs and 3CdS-4ZnS QDNWs. **a**, TEM image of the CdS QDs synthesized in a similar condition with QDNW structure, with the size of 3.2 ± 0.4 nm. The scale bar is 25 nm. **b**, UV-vis absorption spectrum of CdS QDs. The first exciton transition $1S_e$-$1S_h$ of CdS QDs is centered at 424 nm, about 260 meV blue shift compared to $1S_e$-$1S_h$ at 460 nm for 3CdS-4ZnS QDNWs due to a relatively smaller size of the plain CdS QDs. Note that the gradually increased absorbance in the red side results from an inhomogeneous size distribution, consistent with the TA map in (c). **c**,**d**, 2D TA maps of CdS QDs (**c**) and QDNWs (**d**) pumped at 365 nm. The GSB of plain CdS QDs and QDNWs are centered at 424 nm and 460 nm, respectively.

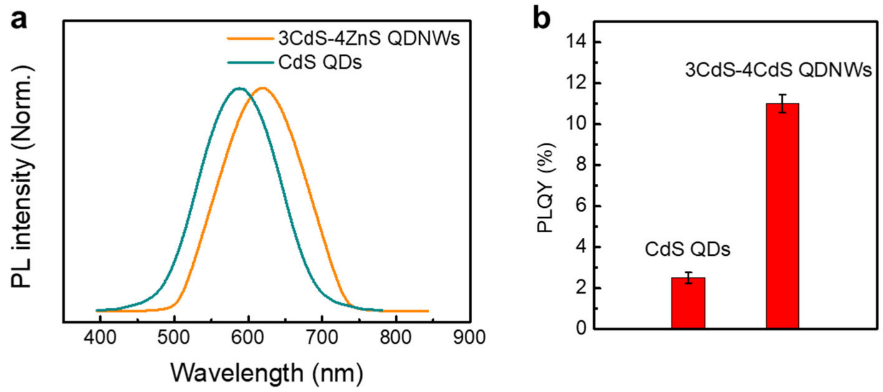

Supplementary Figure 22 | Photoluminesence properties. Photoluminescence spectra (**a**) and absolute photoluminescence quantum yield (PLQY) (**b**) of 3CdS-4ZnS QDNWs and plain CdS QDs. Error bars in **b** correspond to the standard deviation values of PLQY.

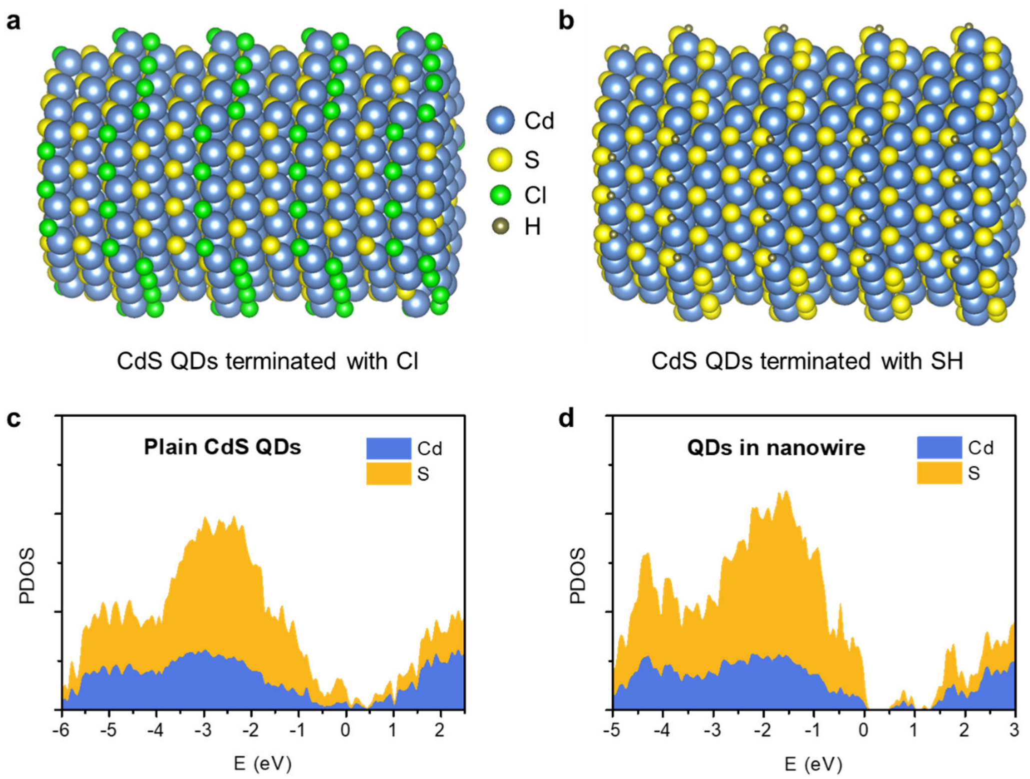

Supplementary Figure 23 | DFT-calculated selective-facet-passivation effect. a,b, Structural models for plain CdS QDs terminated with atomic ligand Cl (**a**) and SH molecules (**b**), respectively. **c**,**d**, DFT-calculated projected density of states for plain CdS QDs (**c**) and CdS QDs in nanowire with (111) facets well passivated (**d**). After (111) facet passivation, trap states are eliminated substantially.

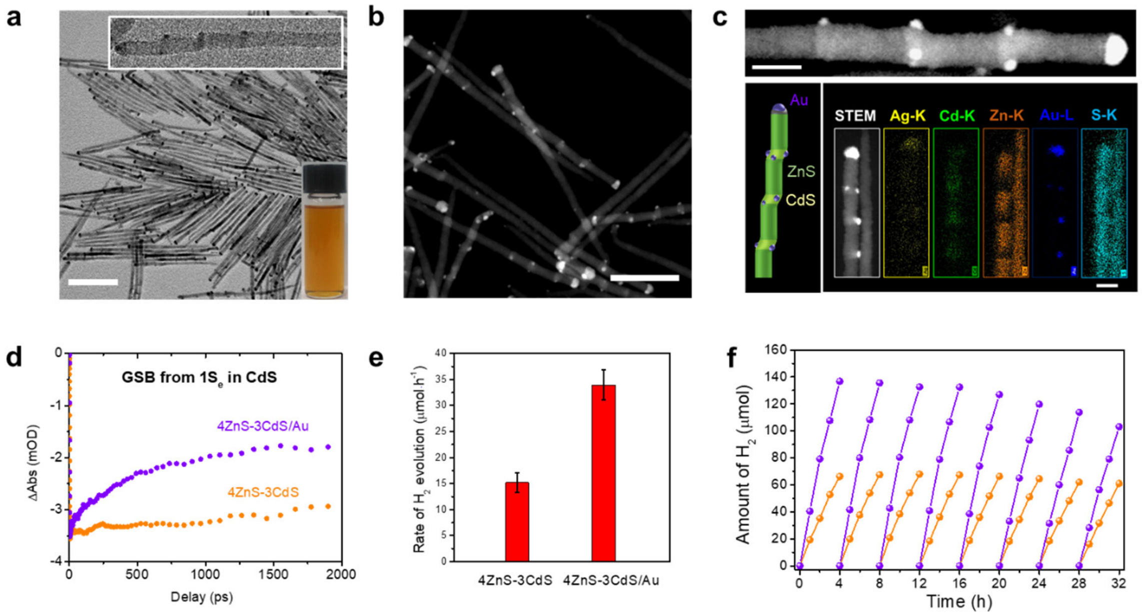

Supplementary Figure 24 | Construction of (3CdS/Au)-4ZnS quantum-dots-in-nanowire with Au selectively decorated on CdS QDs for enhanced charge separation. a, Low-magnification TEM images of (3CdS/Au)-4ZnS with uniform Au nanoparticles decorated on CdS QDs. Inset is corresponding photograph of (3CdS/Au)-4ZnS dispersion in toluene. **b**, Low-magnification (left panel) and a typical enlarged (right panel) dark-field STEM images of (3CdS/Au)-4ZnS. **c**, Dark-field STEM (top panel), geometrical model (left bottom panel) and EDS elemental mapping images (right bottom panel) of the QDNWs. **d**, TA decays (GSB) kinetics of the 3CdS-4ZnS and (3CdS/Au)-4ZnS after 365 nm excitation. **e**, Photocatalytic hydrogen evolution rates of different photocatalysts (20 mg for each). Error bars in **e** correspond to the standard deviation values of H_2 evolution rates. **f**, Recycle stability of photocatalytic performances for 3CdS-4ZnS (yellow) and (3CdS/Au)-4ZnS (violet). Scale bars are 50 nm for **a** and **b**, and 10 nm for **c**, respectively.

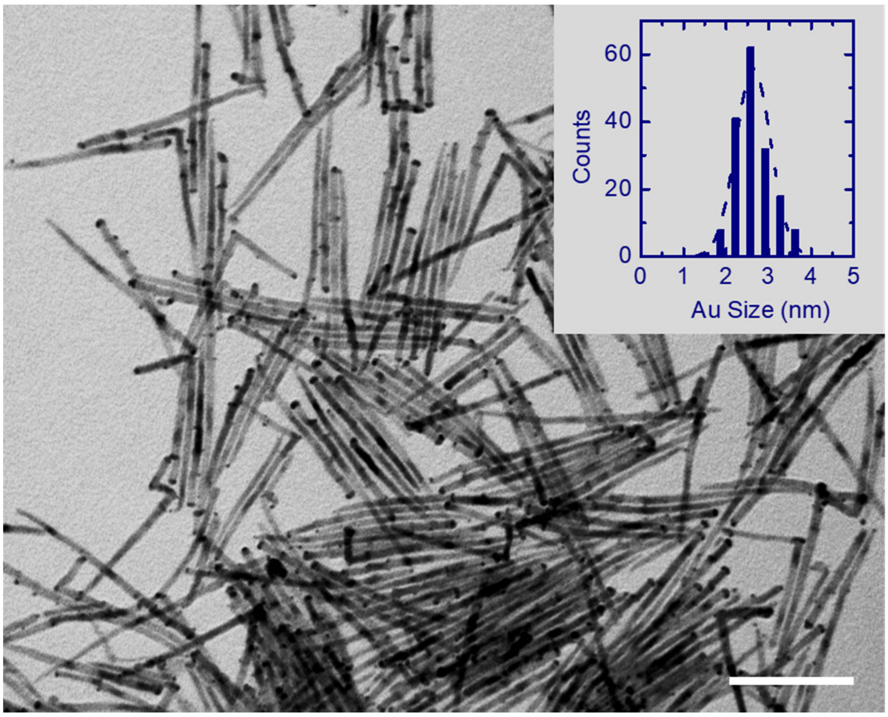

Supplementary Figure 25 | Low magnification TEM image of (3CdS/Au)-4ZnS quantum-dots-in-nanowire. Inset is the size distribution histogram of Au nanoparticles, with the size of 2.6 nm ± 0.4 nm. Scale bar, 100 nm.

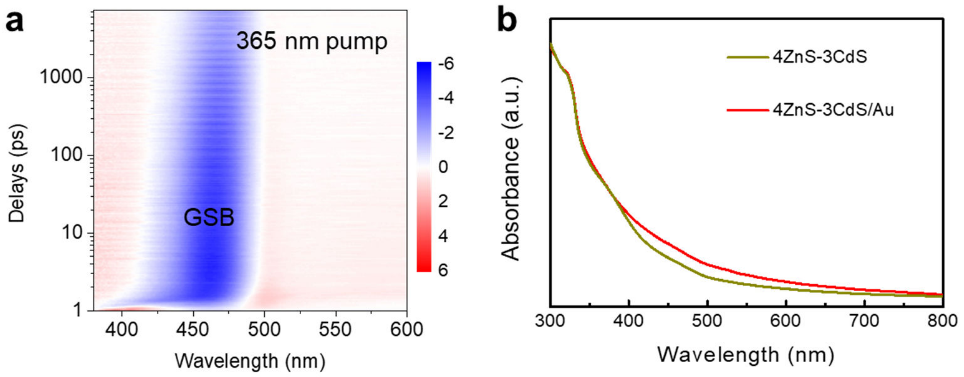

Supplementary Figure 26 | Spectral characterizations of (3CdS/Au)-4ZnS QDNWs. a, 2D TA map of the (3CdS/Au)-4ZnS QDNWs pumped at 365 nm. A clear decay from GSB was observed compared with the 3CdS-4ZnS QDNWs. **b**, UV-vis absorption spectra of the (3CdS/Au)-4ZnS QDNWs.

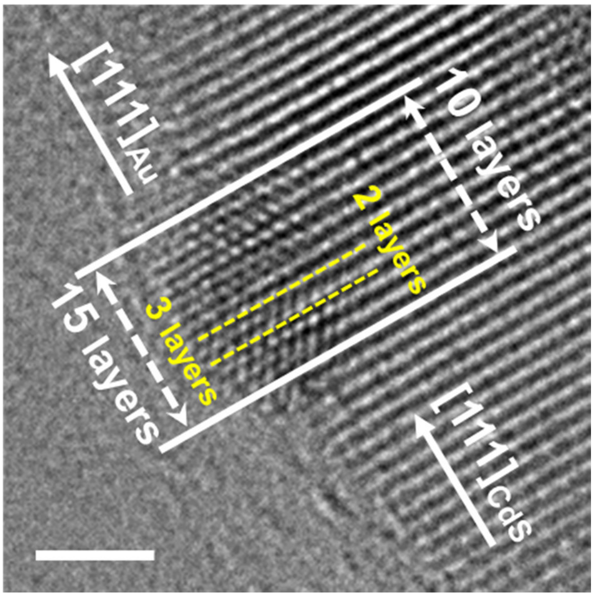

Supplementary Figure 27 | HETEM image of (3CdS/Au)-4ZnS QDNWs.

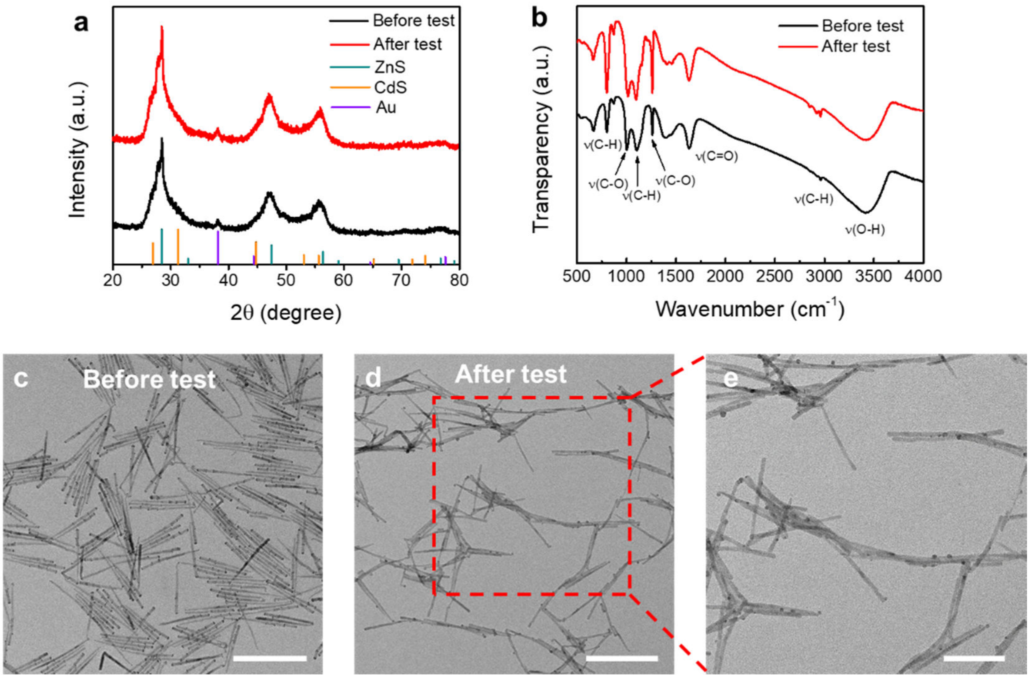

Supplementary Figure 28 | Characterizations of (3CdS/Au)-4ZnS QDNWs before and after photocatalytic stability test. (**a**) XRD patterns before and after reactions. (**b**) FTIR spectra before and after reactions. TEM images before (**c**) and after (**d**, **e**) reactions. Scale bars are 200 nm for **c** and **d**, and 100 nm for **e**, respectively.

Supplementary Table 1 | Comparison of the photocatalytic H_2 production performances for the representative CdS-based photocatalysts with/without cocatalysts.

	Photocatalyst	Amount of photocatalyst (mg)	Sacrificial agents	Temperature (°C)	Light	H_2 production rate (μmol h^{-1})	H_2 production rate (mmol h^{-1} g_{cat}^{-1})	H_2 production rate (mmol h^{-1} g_{CdS}^{-1})	Ref.
Without cocatalyst	CdS nanorods	100	0.35 M Na_2S 0.25 M Na_2SO_3	35	> 430 nm	73.3	0.733	0.733	10
	CdS nanoplates	50	0.35 M Na_2S 0.25 M Na_2SO_3	N/A	> 420 nm	80.5	1.61	1.61	11
	CdS nanoparticles	10	0.1 M Na_2S 0.1 M Na_2SO_3	10	> 420 nm	1.31	0.131	0.131	12
	CdS nanoparticles	20	Lactic acid (22 vol%)	N/A	> 420 nm	2.1	0.105	0.105	13
	CdS NPs	100	Lactic acid (10 vol%)	N/A	> 420 nm	14.8	0.148	0.148	14
	CdS QDs	10	Lactic acid (10 vol%)	N/A	> 420 nm	1.19	0.119	0.119	15
	CdS QDs	100	Lactic acid (20 vol%)	20	> 420 nm	50.8	0.508	0.508	16
	CdS-MPA QDs	0.34	Formic acid (4 M $NaHCO_2$)	25	> 420 nm	17.68	52	52	17
	CdS QDs	100	0.35 M Na_2S 0.25 M Na_2SO_3	15	> 420 nm	173	1.73	1.73	18
	CdS QDs	20	Na_2S + Na_2SO_3	N/A	AM 1.5G	2.32	0.116	0.116	19
	CdS nanoparticles	10	0.1 M Na_2S 0.1 M Na_2SO_3	25	AM 1.5G	3.2	0.32	0.32	20
	CdS nanoparticles	40	Lactic acid (10 vol%)	N/A	> 420 nm	19	0.475	0.475	21
	Cubic CdS	50	0.35 M Na_2S 0.25 M Na_2SO_3	N/A	N/A	360	7.2	7.2	22
	Hexagonal CdS	50	0.35 M Na_2S 0.25 M Na_2SO_3	N/A	N/A	140	2.8	2.8	22
	Hexagonal@cubic CdS nanorods	20	0.35 M Na_2S 0.25 M Na_2SO_3	N/A	> 420 nm	74.2	3.71	3.71	23
	ZB/WZ CdS nanorods	100	0.35 M Na_2S 0.25 M Na_2SO_3	35	> 420 nm	37.6	0.376	0.376	24
	ZB CdS/WZ CdS microrods	100	0.35 M Na_2S 0.25 M Na_2SO_3	N/A	> 420 nm	134	1.34	1.34	25
	CdS nanorods	1	0.75 M Na_2S 1.05 M Na_2SO_3	R.T.	> 420 nm	25	25	25	26
	CdS nanorods	200	Lactic acid (10 vol%)	5	> 420 nm	70	0.35	0.35	27
	CdS-EDTA	1.425	0.25 M Na_2S 0.25 M Na_2SO_3	R.T.	> 420 nm	10.69	7.5	7.5	28
	Ultrathin CdS nanosheets	1.425	0.25 M Na_2S 0.25 M Na_2SO_3	R.T.	> 420 nm	58.57	41.1	41.1	28
	CdS micro/nano leaves	300	0.35 M Na_2S 0.25 M Na_2SO_3	N/A	> 420 nm	741	2.47	2.47	29

	CdS nanowires	40	Lactic acid (10 vol%)	R.T.	> 420 nm	14.8	0.37	0.37	30
	**CdS QDs**	**20**	**0.35 M Na_2S 0.25 M Na_2SO_3**	**20**	**> 420 nm**	**9.2**	**0.46**	**0.54**	**This work**
	CdS-ZnS （$Cd_{0.3}Zn_{0.7}S$ ）	10	0.35 M Na_2S 0.25 M Na_2SO_3	15	> 420 nm	13.4	1.34	4.46	31
	ZnS-CuS-CdS (14.7 wt% Cd)	100	0.06 M Na_2S	N/A	AM 1.5G	83.7	0.837	4.43	32
	CdS-ZnS ($Cd_{0.8}Zn_{0.2}S$)	150	Formic acid (10 vol%)	N/A	> 420 nm	186	1.24	1.55	33
	CdS-mesoporous ZnS	100	0.1 M Na_2S 0.1 M Na_2SO_3	20	> 400 nm	79	0.79	N/A	34
	ZnS-CdS multimode nanorods	20	0.35 M Na_2S 0.25 M Na_2SO_3	R.T.	N/A	12.5	0.675	1.13	35
	ZnS-CdS tetrahedron nanorods	5	0.75 M Na_2S 1.05 M Na_2SO_3	R.T.	> 400 nm	17.2	3.44	3.82	36
	CdS-ZnS-0.5 nanorods	1	0.75 M Na_2S 1.05 M Na_2SO_3	R.T.	> 420 nm	239	239	N/A	37
	ZnO-CdS nanorods	200	0.1 M Na_2S 0.1 M Na_2SO_3	N/A	N/A	592	2.96	11.3	38
	ZnO/CdS	20	Na_2S + Na_2SO_3	N/A	N/A	20	1	1.56	39
	1D porous ZnO/CdS	10	0.5 M Na_2S 0.5 M Na_2SO_3	23	> 400 nm	8.5	0.85	N/A	40
	ZnO/CdS nanourchin	100	0.35 M Na_2S 0.25 M Na_2SO_3	25	> 420 nm	1000	10	N/A	41
	CdS/BiOI	50	Methanol (10 vol%)	N/A	N/A	200	4	N/A	42
	CdS/WO_3	14	Ethanol (50 vol%)	R.T.	> 420 nm	32.6	2.33	2.72	43
	CdS(0.2)/WO_3	50	Lactic acid	N/A	> 400 nm	18.5	0.37	1.85	44
	CdS/CeO_2 nanowires	40	Lactic acid (10 vol%)	R.T.	> 420 nm	21.96	0.549	0.555	30
	CdS(15)@SiO_2 QDs	100	0.35 M Na_2S 0.25 M Na_2SO_3	15	> 420 nm	831	8.31	N/A	18
	**3CdS-4ZnS QDNWs**	**20**	**0.35 M Na_2S 0.25 M Na_2SO_3**	**20**	**> 420 nm**	**15.2**	**0.76**	**5**	**This work**
**With cocatalyst**	CdS/Ni	N/A	NaOH (Ph=14.7)	N/A	447 nm laser (200 mW/cm_2)	N/A	63	N/A	45
	CdS/Ti_3C_2	20	Lactic acid (22 vol%)	N/A	> 420 nm	286.8	14.34	N/A	13
	CdS/Pt	20	Lactic acid (22 vol%)	N/A	> 420 nm	219.6	10.98	N/A	13
	CdS/Pd	1	Lactic acid (20 vol%)	N/A	AM 1.5G	130.33	130.33	N/A	46
	CdS/Pt/PdS	300	0.35 M Na_2S 0.25 M Na_2SO_3	12-18	> 420 nm	8760	29.2	N/A	47
	CdS/MoS_2	100	Lactic acid (10 vol%)	N/A	> 420 nm	520	5.2	N/A	14
	CdS/MoS_2	200	Lactic acid (10 vol%)	5	> 420 nm	12056	60.28	N/A	27

CdS/MoS_2	10	Lactic acid (10 vol%)	N/A	> 420 nm	19.8	1.98	N/A	15
CdS/CoP	20	Lactic acid (10 vol%)	5	> 420 nm	2120	106	N/A	48
**(3CdS/Au)-4ZnS QDNWs**	**20**	**0.35 M Na_2S 0.25 M Na_2SO_3**	**20**	**> 420 nm**	**34**	**1.7**	**10.67**	**This work**

Note that only semiconductors with larger or comparable bandgaps than that of CdS are listed here.

Supplementary Table 2 | Fitted ground state transition energies of ZnS and CdS segments.

	G11 (CdS)	G12 (CdS)	G13 (CdS)	G14 (CdS)	G21 (ZnS)	G22 (ZnS)
E_i (eV) /λ	2.70 (460 nm)	2.82 (440 nm)	3.08 （403 nm）	3.40 (365 nm)	3.95 (315 nm)	4.14 (310 nm)

Supplementary Note 1: Phase transitions in pulsed axial epitaxy

In VLS growth, the low supersaturation in catalyst is prone to yield ZB crystal structure. Consistently, ZB structure dominates in our QDNWs, due to the very low supersaturation of Zn/Cd in Ag_2S solid catalyst.

The observed two epitaxy modes, ZB-ZB(TW)-WZ and WZ-ZB-WZ, could be explained as below[49] (**Supplementary Fig.10)**: First, in the initial growth stage of ZnS nanowire, the supersaturation of ZnS ($\Delta\mu_{SS}^{ZnS}$) is below but close to its critical supersaturation ($\Delta\mu_{c}^{ZnS}$), which determines the ZB or WZ crystalline phase. Due to the small formation energy differences between ZB and WZ phase, thermal fluctuations could give occasionally rise to WZ ZnS. When Cd precursors are introduced, the higher solubility of Cd in Ag_2S catalyst leads to increased $\Delta\mu_{SS}^{CdS}$ compared to $\Delta\mu_{SS}^{ZnS}$, but still lower than $\Delta\mu_{c}^{CdS}$ and thus crystallize in the form of ZB CdS. Finally, once Cd precursors are depleted, ZnS regrows again along the nanowire. At the initial regrowth stage, accumulated Zn atoms in catalyst lead to a higher $\Delta\mu_{SS}^{ZnS}$ than that in stable regrowth stage, thereby the crystalline phase of ZnS segment transforms from WZ to ZB.

Note that, the occurrence of two epitaxy modes is theoretically random, because the introduction time of Cd precursor is absolutely random. Once the Cd precursor is added to the reaction solution, a burst of Cd concentration would lead to prevailing epitaxy of CdS segment. The introduction time of Cd precursor is up to individuals on the time scale of minutes. However, we observed the dominance of ZB-ZB (TW)-WZ epitaxy mode in the quantum-dots-in-nanowire structure. This could be attributed to the dominance of zinc-blend structure during the growth of ZnS NRs. No distinguishable peak indexed to (311) plane of wurtzite structure can be observed in the typical XRD pattern.

Supplementary Note 2: Effect of temperature on nanowire diameter and length

The nanowire diameter and length are highly dependent on the reaction temperature:

(1) Temperature-diameter relationship

The reaction temperature determines the initial size of Ag_2S catalyst which nucleates first in our one-pot synthesis. Higher reaction temperature results in larger catalyst size and consequently thicker nanowires. Therefore, we can modulate the nanowire diameter by simply changing the reaction temperature.

(2) Temperature-length relationship

In the one-pot reaction, we note that, QDNWs with larger diameter grow faster. This is consistent with the dominant role of aforementioned Gibbs-Thomson effect – larger diameter results in higher supersaturation and faster growth, hence longer nanowires[50,51]. Note that, although the enhanced growth rate can not be simply distinguished between diameter contribution and the additional influence of temperature (higher reaction rate at higher temperature), we did observe higher growth rate with larger catalyst when we used large Ag_2S nanoparticles (nucleated at 250 ^oC) to catalyze the nanowire growth at low temperature (210 ^oC).

Supplementary Note 3: Determination of band structures

The absorption spectra of ZnS and CdS segment were fitted with two and four Gaussian peaks, respectively, for ground state transitions. Fit functions are in the form:

$$f(\lambda) = \sum_i \frac{A_i}{\sqrt{2\pi\sigma_i^2}} \exp\left[-\left(\lambda - \lambda_{0,i}\right)^2 / 2\sigma_i^2\right] \quad (1)$$

Fitted Gaussian peaks are shown in **Supplementary Fig. 20** and fitted parameters are listed in **Supplementary Table. 2**.

Supplementary Note 4: Site-selective nucleation of Au nanoparticles on CdS

Lattice matching is responsible for the site-selective nucleation of Au NPs on CdS QDs. The d spacings of CdS (111) planes, ZnS (111) planes, and Au (111) planes are 0.335, 0.311, and 0.235 nm, respectively. The lattice mismatch between 3 layers of Au (111) plane and 2 layers of CdS (111) plane is 5.2%. In contrast, the lattice mismatch between the ZnS and Au is 13.3 %. The HRTEM image of (3CdS/Au)-4ZnS QDNWs, as shown in **Supplementary Fig. 27**, accords well with our calculations. 10 layers of CdS (111) planes match well with 15 layers of Au (111) planes.

Supplementary Note 5: Analysis on the photocatalytic stability degradation

We characterized the sample of (3CdS/Au)-4ZnS before and after the photocatalytic stability test to understand the causes of performance degradation. There is no difference in the XRD patterns (**Supplementary Fig. 28a**) and FTIR spectra (**Supplementary Fig. 28b**) for both samples. However, we note in TEM images that the Au nanoparticles (NPs) become larger and inhomogeneously distributed after stability test (**Supplementary Fig. 28c-e**).

We attribute this to Ostwald ripening, corresponding to lowering of the total surface Gibbs free energy. A similar transformation from Au clusters to Au nanoparticles, and an analogous

performance degradation, were also observed by Xu et al[52]. Thus, the ripening of Au NPs and the losses of Au NPs on parts of CdS segments induced the performance degradation.

Supplementary Methods

Chemicals

$AuCl_3$ (50%), potassium hydroxide (KOH), sodium sulfite (Na_2SO_3), hexane (97%), toluene (99.5%), methanol (99.5%), ethanol(99.7%), 1-dodecanethiol (DDT, 97%), oleic acid (OA, 85%) were purchased from the Shanghai Reagent Company (P. R. China). Oleylamine (OAm, 80-90%) and sodium sulfide nonahydrate ($Na_2S{\cdot}9H_2O$) were purchased from Aladdin Chemicals. All chemicals were used as received without further purification.

Synthesis of CdS-ZnS quantum-dots-in-nanowire

The ultrathin 3CdS-4ZnS QDNWs were synthesized via a catalyst-assisted method. Typically, Ag(dedc) (14.3 mg) and $Zn(dedc)_2$ (235.5 mg) were added into 10 mL DDT in a three-neck flask and heated to 210 ^oC in 22 min with magnetic stirring. Then 10 mL OA was slowly injected after keeping the solution at 210 ^oC for 5 min. The reaction mixture color turned turbid gray. After initial ZnS growth for a certain time (30-60 min), 40 mg $Cd(dedc)_2$ was added *in situ* into the reaction for three times with a time interval of 20 min. An aliquot of products in each stage was taken and precipitated with ethanol for analyses. The final QDNWs were collected and centrifuged. Then, the products were washed twice with hexane and ethanol for further use.

The procedure for growing CdS-ZnS QDNWs with different diameters is similar with that of 3CdS-4ZnS QDNWs, except that the reaction temperature was elevated to 190-250 ^oC (determined by the nanowire diameter we need) before 10 mL OA was slowly injected. Note that, for nanowires synthesized at 250 ^oC, the initial ZnS growth time was reduced to 30 min due to a relatively higher consumption rate of Zn precursor in solution.

The procedure for growing CdS-ZnS (ZnS elongated) QDNWs is similar to that of CdS-ZnS QDNWs, except that 40 mg $Cd(dedc)_2$ and 50 mg $Zn(dedc)_2$ was alternatively added into the reaction with time interval of 20 min after the initial ZnS growth.

The procedure for growing CdS-ZnS (CdS elongated) QDNWs is similar to that of CdS-ZnS QDNWs, except another 40 mg $Cd(dedc)_2$ was added into the reaction after 4 min growth of the

first CdS segment and this process was repeated again every 20 min. Each CdS segment is roughly estimated to be 8 nm.

Supplementary References

1. Wu, K., Li, Q., Du, Y., Chen, Z., Lian, T., Ultrafast exciton quenching by energy and electron transfer in colloidal CdSe nanosheet-Pt heterostructures. *Chem. Sci.* **6**, 1049-1054 (2015).
2. Wu, K., Zhu, H., Liu, Z., Rodríguez-Córdoba, W., Lian, T., Ultrafast charge separation and long-lived charge separated state in photocatalytic CdS–Pt nanorod heterostructures. *J. Am. Chem. Soc.* **134**, 10337-10340 (2012).
3. Utterback, J. K. et al., Observation of trapped-hole diffusion on the surfaces of CdS nanorods. *Nat. Chem.* **8**, 1061-1066 (2016).
4. Wu, K., Lian, T., Quantum confined colloidal nanorod heterostructures for solar-to-fuel conversion. *Chem. Soc. Rev.* **45**, 3781-3810 (2016).
5. Pal, B. N. *et al.*, 'Giant' CdSe/CdS core/shell nanocrystal quantum dots as efficient electroluminescent materials: strong influence of shell thickness on light-emitting diode performance. *Nano Lett.* **12**, 331-336 (2012).
6. Stier, A. V., Wilson, N. P., Clark, G., Xu, X., Crooker, S. A., Probing the influence of dielectric environment on excitons in monolayer WSe_2: insight from high magnetic fields. *Nano Lett.* **16**, 7054-7060 (2016).
7. Komsa, H.-P., Krasheninnikov, A. V., Effects of confinement and environment on the electronic structure and exciton binding energy of MoS_2 from first principles. *Phys. Rev. B* **86**, 241201 (2012).
8. Christodoulou, S. *et al.*, Band structure engineering via piezoelectric fields in strained anisotropic CdSe/CdS nanocrystals. *Nat. Commun.* **6**, 7905 (2015).
9. McDonald, M. P., Chatterjee, R., Si, J., Janko, B., Kuno, M., Dimensional crossover in semiconductor nanostructures. *Nat. Commun.* **7**, 12726 (2016).
10. Liu, M., Jing, D., Zhou, Z., Guo, L., Twin-induced one-dimensional homojunctions yield high quantum efficiency for solar hydrogen generation. *Nat. Commun.* **4**, 2278 (2013).
11. Wang, X., Liu, M., Zhou, Z., Guo, L., Toward facet engineering of CdS nanocrystals and their shape-dependent photocatalytic activities. *J. Phys. Chem. C* **119**, 20555-20560 (2015).

12. Chen, Y. *et al.*, Synergetic integration of $Cu_{1.94}S$-$Zn_xCd_{1-x}S$ heteronanorods for enhanced visible-light-driven photocatalytic hydrogen production. *J. Am. Chem. Soc.* **138**, 4286-4289 (2016).
13. Ran, J. *et al.*, Ti_3C_2 MXene co-catalyst on metal sulfide photo-absorbers for enhanced visible-light photocatalytic hydrogen production. *Nat. Commun.* **8**, 13907 (2017).
14. Zong, X. *et al.*, Enhancement of Photocatalytic H_2 Evolution on CdS by Loading MoS_2 as Cocatalyst under Visible Light Irradiation. *J. Am. Chem. Soc.* **130**, 7176-7177 (2008).
15. Chen, J. *et al.*, One-pot synthesis of CdS nanocrystals hybridized with single - layer transition - metal dichalcogenide nanosheets for efficient photocatalytic hydrogen evolution. *Angew. Chem. Int. Ed.* **54**, 1210-1214 (2015).
16. Zha, D.-W., Li, L.-F., Pan, Y.-X., He, J.-B., Coconut shell carbon nanosheets facilitating electron transfer for highly efficient visible-light-driven photocatalytic hydrogen production from water. *Int. J. Hydrogen Energy* **41**, 17370-17379 (2016).
17. Kuehnel, M. F., Wakerley, D. W., Orchard, K. L., Reisner, E., Photocatalytic Formic Acid Conversion on CdS Nanocrystals with Controllable Selectivity for H_2 or CO. *Angew. Chem. Int. Ed.* **54**, 9627-9631 (2015).
18. Yu, G., Geng, L., Wu, S., Yan, W., Liu, G., Highly-efficient cocatalyst-free H_2-evolution over silica-supported CdS nanoparticle photocatalysts under visible light. *Chem. Commun.* **51**, 10676-10679 (2015).
19. Qiu, B. et al., Efficient Solar Light Harvesting CdS/Co_9S_8 Hollow Cubes for Z-Scheme Photocatalytic Water Splitting. *Angew. Chem. Int. Ed.* **56**, 2684-2688 (2017).
20. Xing, M. et al., Spatially Separated CdS Shells Exposed with Reduction Surfaces for Enhancing Photocatalytic Hydrogen Evolution. *Adv. Funct. Mater.* **27**, 1702624 (2017).
21. Yang, M.-Q., Han, C., Xu, Y.-J., Insight into the Effect of Highly Dispersed MoS_2 versus Layer-Structured MoS_2 on the Photocorrosion and Photoactivity of CdS in Graphene–CdS–MoS_2 Composites. *J. Phys. Chem. C* **119**, 27234-27246 (2015).
22. Yu, H., Zhong, W., Huang, X., Wang, P., Yu, J., Suspensible Cubic-Phase CdS Nanocrystal Photocatalyst: Facile Synthesis and Highly Efficient H_2-Evolution Performance in a Sulfur-Rich System. *ACS Sustainable Chem. Eng.* **6**, 5513-5523 (2018).
23. Li, K. et al., Hexagonal@cubic CdS core@shell nanorod photocatalyst for highly active production of H_2 with unprecedented stability. *Adv. Mater.* **28**, 8906-8911 (2016).

24. He, K., Guo, L., A-Novel-CdS-Nanorod with Stacking Fault Structures: Preparation and Properties of Visible-Light-Driven Photocatalytic Hydrogen Production from Water Splitting. *Energy Procedia* **61**, 2450-2455 (2014).
25. Liu, M. *et al.*, Transformation of zincblende nanoparticles into wurtzite microrods by a dissolution-regrowth process: an intergrowth homojunction with enhanced photocatalytic activity. *Catal. Sci. & Technol.* **6**, 3371-3377 (2016).
26. Sun, Z., Zheng, H., Li, J., Du, P., Extraordinarily efficient photocatalytic hydrogen evolution in water using semiconductor nanorods integrated with crystalline Ni_2P cocatalysts. *Environ. Environ. Sci.* **8**, 2668-2676 (2015).
27. Yin, X.-L. et al., Rational design and electron transfer kinetics of MoS_2/CdS nanodots-on-nanorods for efficient visible-light-driven hydrogen generation. *Nano Energy* **28**, 319-329 (2016).
28. Xu, Y., Zhao, W., Xu, R., Shi, Y., Zhang, B., Synthesis of ultrathin CdS nanosheets as efficient visible-light-driven water splitting photocatalysts for hydrogen evolution. *Chem. Commun.* **49**, 9803-9805 (2013).
29. Li, C. et al., Controlled synthesis of CdS micro/nano leaves with (0001) facets exposed: enhanced photocatalytic activity toward hydrogen evolution. *J. Mater. Chem.* **22**, 23815-23820 (2012).
30. Zhang, X., Zhang, N., Xu, Y.-J., Tang, Z.-R., One-dimensional CdS nanowires–CeO_2 nanoparticles composites with boosted photocatalytic activity. *New J. Chem.* **39**, 6756-6764 (2015).
31. Kai, S. *et al.*, An innovative Au-CdS/ZnS-RGO architecture for efficient photocatalytic hydrogen evolution. *J. Mater. Chem. A* **6**, 2895-2899 (2018).
32. Hong, E., Kim, D., Kim, J. H., Heterostructured metal sulfide (ZnS–CuS–CdS) photocatalyst for high electron utilization in hydrogen production from solar water splitting. *J. Ind. Eng. Chem.* **20**, 3869-3874 (2014).
33. Wang, X., Li, X.-Y., Heterostructure CdS/ZnS nanoparticles as a visible light-driven photocatalyst for hydrogen generation from water. *Int. J. Green Energy* **13**, 1201-1208 (2016).
34. Xie, Y. P., Yu, Z. B., Liu, G., Ma, X. L., Cheng, H.-M., CdS-mesoporous ZnS core-shell particles for efficient and stable photocatalytic hydrogen evolution under visible light.

Environ. Environ. Sci. **7**, 1895-1901 (2014).

35. Zhuang, T. T., *et al.*, A unique ternary semiconductor-(semiconductor/metal) nano-architecture for efficient photocatalytic hydrogen evolution. *Angew. Chem. Int. Ed.* **54**, 11495-11500 (2015).

36. Zhuang, T. T., *et al.*, 1D Colloidal hetero-nanomaterials with programmed semiconductor morphology and metal location for enhancing solar energy conversion. *Small* **13**, 1602629 (2017).

37. Jiang, D., Sun, Z., Jia, H., Lu, D., Du, P., A cocatalyst-free CdS nanorod/ZnS nanoparticle composite for high-performance visible-light-driven hydrogen production from water. *J. Mater. Chem. A* **4**, 675-683 (2016).

38. Wang, X., Liu, G., Lu, G. Q., Cheng, H.-M., Stable photocatalytic hydrogen evolution from water over ZnO–CdS core–shell nanorods. *Int. J. Hydrogen Energy* **35**, 8199-8205 (2010).

39. Mukhopadhyay, S., Mondal, I., Pal, U., Devi, P. S., Fabrication of hierarchical ZnO/CdS heterostructured nanocomposites for enhanced hydrogen evolution from solar water splitting. *Phys. Chem. Chem. Phys.* **17**, 20407-20415 (2015).

40. Zou, X. et al., One-pot cation exchange synthesis of 1D porous CdS/ZnO heterostructures for visible-light-driven H_2 evolution. *J. Mater. Chem. A* **2**, 4682-4689 (2014).

41. Barpuzary, D., Khan, Z., Vinothkumar, N., De, M., Qureshi, M., Hierarchically Grown Urchinlike CdS@ZnO and CdS@Al_2O_3 Heteroarrays for Efficient Visible-Light-Driven Photocatalytic Hydrogen Generation. *J. Phys. Chem. C* **116**, 150-156 (2012).

42. Kandi, D., Martha, S., Thirumurugan, A., Parida, K. M., Modification of BiOI Microplates with CdS QDs for Enhancing Stability, Optical Property, Electronic Behavior toward Rhodamine B Decolorization, and Photocatalytic Hydrogen Evolution. *J. Phys. Chem. C* **121**, 4834-4849 (2017).

43. Huang, Y., Liu, Y., Zhu, D., Xin, Y., Zhang, B., Mediator-free Z-scheme photocatalytic system based on ultrathin CdS nanosheets for efficient hydrogen evolution. *J. Mater. Chem. A* **4**, 13626-13635 (2016).

44. Zhang, L. J., Li, S., Liu, B. K., Wang, D. J., Xie, T. F., Highly Efficient CdS/WO_3 Photocatalysts: Z-Scheme Photocatalytic Mechanism for Their Enhanced Photocatalytic H_2 Evolution under Visible Light. *ACS Catalysis* **4**, 3724-3729 (2014).

45. Simon, T. et al., Redox shuttle mechanism enhances photocatalytic H_2 generation on Ni-decorated CdS nanorods. *Nat. Mater.* **13**, 1013 (2014).
46. Park, H. et al., Synthesis of Ultra-Small Palladium Nanoparticles Deposited on CdS Nanorods by Pulsed Laser Ablation in Liquid: Role of Metal Nanocrystal Size in the Photocatalytic Hydrogen Production. *Chem. – A Eur. J.* **23**, 13112-13119 (2017).
47. Yan, H. et al., Visible-light-driven hydrogen production with extremely high quantum efficiency on Pt–PdS/CdS photocatalyst. *J. Catal.* **266**, 165-168 (2009).
48. Zhao, D. et al., Promoting visible light-driven hydrogen evolution over CdS nanorods using earth-abundant CoP as a cocatalyst. *RSC Adv.* **6**, 33120-33125 (2016).
49. Dheeraj D. L. et al., Growth and Characterization of Wurtzite GaAs Nanowires with Defect-Free Zinc Blende GaAsSb Inserts. *Nano Lett.* **8**, 4459-4463 (2008).
50. Laocharoensuk, R. *et al.*, Flow-based solution–liquid–solid nanowire synthesis. *Nat. Nanotechnol.* **8**, 660-666 (2013).
51. Givargizov, E. I. Fundamental aspects of VLS growth, J. Crys. Growth 31, 20-30 (1975).
52. Liu, S., Xu, Y.-J., Photo-induced transformation process at gold clusters-semiconductor interface: Implications for the complexity of gold clusters-based photocatalysis. *Sci. Rep.* **6**, 22742 (2016).
